# Mapping Worldwide Antibiotic Use in Dental Practices: A Scoping Review

**DOI:** 10.3390/antibiotics13090859

**Published:** 2024-09-08

**Authors:** Fatemeh Soleymani, Carlos Pérez-Albacete Martínez, Mehrdad Makiabadi, José Eduardo Maté Sánchez de Val

**Affiliations:** 1 Health Sciences PhD Program, UCAM-Universidad Católica San Antonio de Murcia, Campus de los Jerónimos nº135, Guadalupe, 30107 Murcia, Spain; 2 Department of Biomaterials Engineering, Faculty of Health Sciences, UCAM-Universidad Católica San Antonio de Murcia, Guadalupe, 30107 Murcia, Spain; cperezalbacete@ucam.edu (C.P.-A.M.); jemate@ucam.edu (J.E.M.S.d.V.); 3 Independent Researcher, 30007 Murcia, Spain

**Keywords:** antibiotic resistance, defined daily doses, dentistry

## Abstract

Antibiotic resistance is a critical issue today, necessitating the monitoring of antibiotic usage across various sectors. To determine the defined daily doses (DDDs) of antibiotics prescribed by dentists globally, a comprehensive search was conducted in PubMed, ProQuest, ScienceDirect, Web of Science, Scopus, and EBSCOhost, resulting in the inclusion of 15 documents in this scoping review. The DDD per 1000 inhabitants per day (DID) for oral antibiotics prescribed by dentists for the studied countries was generally below 2.11, with the exception of South Korea, which had a DDD per 1000 patients per day (DPD) of less than 6.97. Most countries, except Croatia and Belgium, demonstrated a decreasing trend in DID before the COVID-19 pandemic, but restrictions during the pandemic led to an increase in these numbers. Penicillin-derived antibiotics were the most commonly prescribed antibiotic among dentists in most countries. This study highlights significant gaps and missing data regarding antibiotics prescribed by dentists worldwide. However, it also indicates that the publication of guidelines, education, and evaluation of antibiotic use can lead to more controlled and appropriate prescriptions among dental professionals.

## 1. Introduction

Antibiotics are crucial in infection treatment and prevention. However, the World Health Organization (WHO) report presented a significant threat to public health worldwide caused by resistance rates among prevalent bacterial pathogens [1]. In 2019, antimicrobial-resistant bacteria were estimated to be directly responsible for approximately 1.27 million deaths globally [2]. If current trends continue, this number could rise to 10 million deaths annually by 2050 [3]. Although antibiotic resistance is a natural process in bacteria that happens through genetic mutations, human activity, especially misuse and overuse of antimicrobial agents to treat, prevent, or control infective diseases in humans, animals, and plants, increases the speed of this process [4]. This alarming increase underscores the urgent need for comprehensive strategies to combat antibiotic resistance.

In the field of dentistry, antibiotics are frequently prescribed to prevent and treat infections, especially for odontogenic abscesses, pulp and preapical tissue diseases, chronic apical periodontitis, and medication-related osteonecrosis of the jaw (MRONJ) [5,6]. Dentists are among the top prescribers of antibiotics, accounting for about 10% of all outpatient antibiotic prescriptions [7]. Based on the reports, the most commonly used antibiotics in the field of dentistry include amoxicillin, clindamycin, penicillin VK, azithromycin, metronidazole, and amoxicillin–clavulanate [5,8,9,10,11,12]. Investigations indicated that a considerable number of antibiotic prescriptions by dentists are either unnecessary or inappropriate. For instance, 27.8% of antibiotics prescribed in the Albaha Region of Saudi Arabia were found to be inaccurate [13]. In the United States, despite a downward trend, 14% of antibiotic prescriptions by general dentists were still inappropriate [14]. In Croatia, less than half of antibiotics prescribed between 2015 and 2019 were for correct indications [5]. Furthermore, in Lebanon, the exclusive prescription of broad-spectrum antibiotics for apical abscesses has raised significant concerns about antibiotic resistance [15].

The WHO defines defined daily doses (DDDs) as the assumed average maintenance dose per day for a drug used for its main indication in adults. DDDs are calculated based on the average dose recommended for the drug’s primary indication in adults. This involves reviewing clinical guidelines, the literature, and expert opinions to determine a single recommended dose or an average dose from a range of recommended doses. The DDD is then assigned an Anatomical Therapeutic Chemical (ATC) classification code, which groups drugs based on their therapeutic use and chemical characteristics. This metric allows for the comparison of drug usage across different regions and time periods, facilitating better understanding and management of drug consumption patterns. It is particularly useful in monitoring trends in drug use, assessing the impact of interventions, and guiding policy decisions [16]. Each year, the WHO releases data on DDDs per 1000 inhabitants per day (DID) globally through the Global Antimicrobial Resistance and Use Surveillance System (GLASS) project. These reports highlight changes in antibiotic DDDs across different countries over time. However, they do not provide separate data specifically for dentistry [1].

The objective of this study is to conduct a scoping review of antibiotic prescriptions within the field of dentistry. This review aims to answer the following question: “What is the Defined daily doses of antibiotic prescribed by dentists in each country?” To achieve this, we employed a broad search strategy across multiple databases and included various sources such as government reports and direct communications with authors. Additionally, this study seeks to develop a global map illustrating the DDDs per 1000 inhabitants per day (DID) for antibiotics across different countries. This map underscores the critical importance of understanding antibiotic prescribing patterns and highlights significant gaps in the existing data. By mapping these patterns, this study provides valuable insights into the global landscape of dental antibiotic use and emphasizes the need for more detailed and accurate data collection in this area. As a secondary aim, the extracted data are used to reveal the most frequently prescribed antibiotics by dental professionals in various countries and to highlight trends in antibiotic prescriptions before, during, and after the COVID-19 pandemic.

## 2. Results

**Figure 1:**
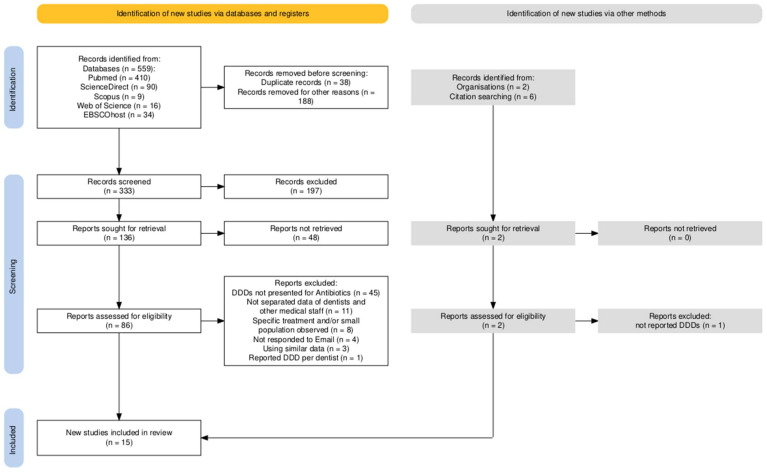
PRISMA flowchart for included studies and details of excluded ones, created using PRISMA Flow Diagram tool [17].

A meticulous search was conducted across specified databases and supplemented by a manual examination of references from related systematic reviews. This search yielded 559 articles. Upon initial screening, 38 and 188 articles were excluded due to duplication and irrelevance as determined by their titles, respectively. Subsequent to these exclusions, 333 articles underwent abstract screening. This phase resulted in the removal of 197 articles due to the established exclusion criteria and an additional 48 articles that reported data prior to 2014. A detailed full-text review of the remaining 86 articles led to the exclusion of 45 articles, which failed to provide sufficient information for the calculation of DDDs or DID for dental practitioners. The exclusion reasons are elaborated in Figure 1, which depicts the PRISMA flowchart of the article selection process, constructed using the PRISMA Flow Diagram tool [17]. In the final stage, 15 documents were selected for data extraction and map construction, consisting of 13 provisional articles [5,8,9,18,19,20,21,22,23,24,25,26,27], 1 governmental report [28], and 1 systematic review [29]. The systematic review [29] was included due to its comprehensive search of governmental websites for records of antibiotic use in dentistry within Germany, aligning with the objectives of this scoping review.

### 2.1. Risk-of-Bias Assessment

**Figure 2:**
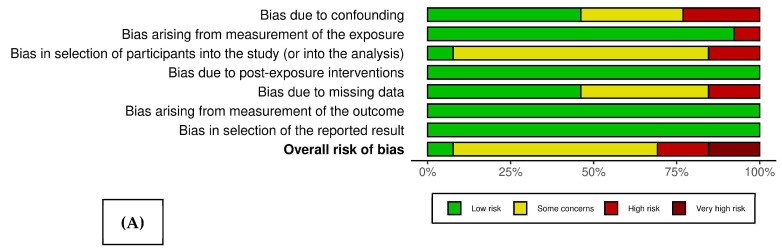

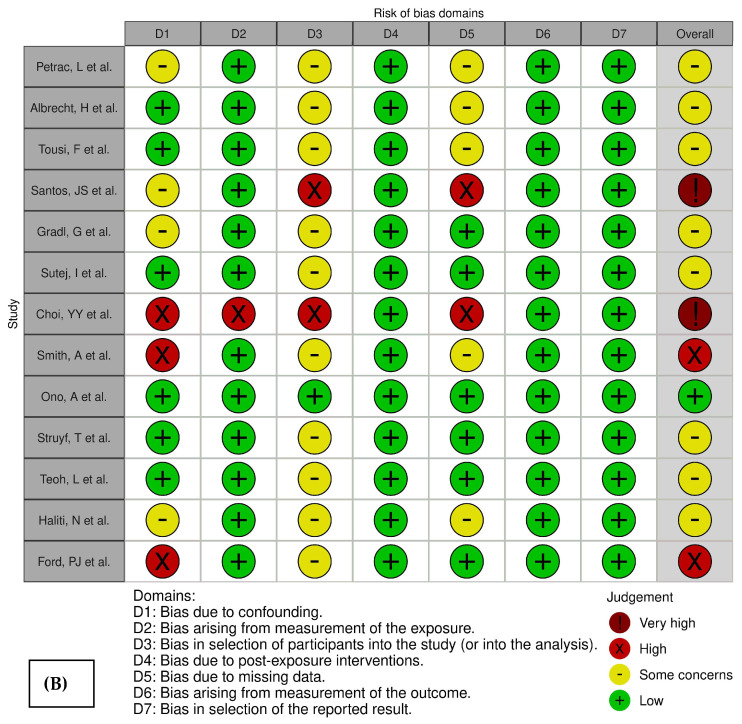
The weighted bar plot (**A**) and traffic light plot (**B**), which delineate the bias risk across the 13 studies under consideration [5,8,9,18,19,20,21,22,23,24,25,26,27].

Figure 2 presents the weighted bar plot (A) and traffic light plot (B), which delineate the bias risk across the 13 studies under consideration [5,8,9,18,19,20,21,22,23,24,25,26,27]. Notably, the analysis included a governmental report [28] and a systematic review [29], neither of which are amenable to bias risk evaluation via the ROBINS-E framework. With the exception of one study [23] that was determined to have a low risk of bias, the remaining studies exhibited varying degrees of concern. This was particularly evident within the domain of selection bias, attributable to the inherent nature of their database sources, as detailed in Table 1.

### 2.2. Detailed Extracted Data

Table 1 outlines the details of each study, containing the countries analyzed, the databases employed along with their particulars, the time frames of the databases, the type of antibiotic prescriptions (therapeutic and/or prophylactic), the categories of dental practitioners administering prescriptions (dentists and/or dental specialists), the most frequently prescribed antibiotics by dental professionals within the study’s time frame, and any available trends in antibiotic prescription by dental staff. It is observed that the majority of databases incorporate data from public centers or individuals covered by insurance policies, omitting antibiotics prescribed to hospitalized patients. This exclusion results in a piece of missing data, leading to inevitable selection bias. Notably, the study that includes data from South Korea [21] centered on patients who had undergone tooth extractions, and the DID provided was calculated based on 1000 patients, rather than 1000 general population. Despite this limitation, this study was included in the study as it represents the only data founded from the specified country.

### 2.3. Most Prescribed Antibiotics by Dentists in Each Country

Table 1 reveals that amoxicillin was the antibiotic most frequently prescribed by dental professionals in England [22,28], Brazil [18], Germany [19], Scotland [22], and Australia [25,27]. In Croatia [5,20] and Kosovo [26], amoxicillin with clavulanic acid was the preferred choice, whereas in Germany, oral penicillin and aminopenicillins such as amoxicillin were predominantly prescribed [8,29]. In Sweden [22] and Norway [9,22], phenoxymethylpenicillin was the common prescription, while cephalosporins were favored by dentists in Japan [23]. The use of broad-spectrum antibiotics and amoxicillin, with or without an enzyme inhibitor, was more prevalent in South Korea and Belgium, respectively [21,24].

### 2.4. Mapping Antibiotic Prescription in Dentistry around the World

**Figure 3:**
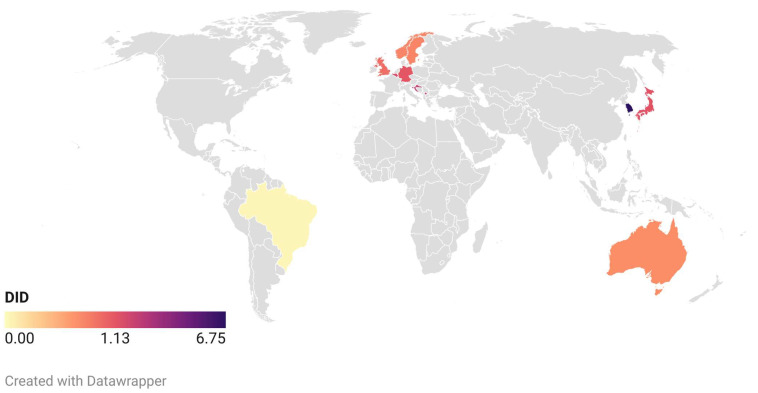
Global map of defined daily doses per 1000 inhabitants per day for antibiotics prescribed by dentists, based on founded information.

The antibiotic prescription data for dental professionals from the year 2016 were instrumental in constructing the global map depicted in Figure 3 (data available in Table 2). The data for Kosovo correspond to the year 2015, while the Brazilian data are from 2017. The map reveals that, despite extensive searches, applicable data for most countries remained elusive or unavailable up to the point of this research. However, the encountered data demonstrate that the minimum DID belongs to Brazil [18] with 0.05, and the maximum DID is for South Korea (6.09–6.97) [21].

### 2.5. Antibiotic Prescription Trends by Dentists before, during, and after COVID-19 Pandemic

**Figure 4:**
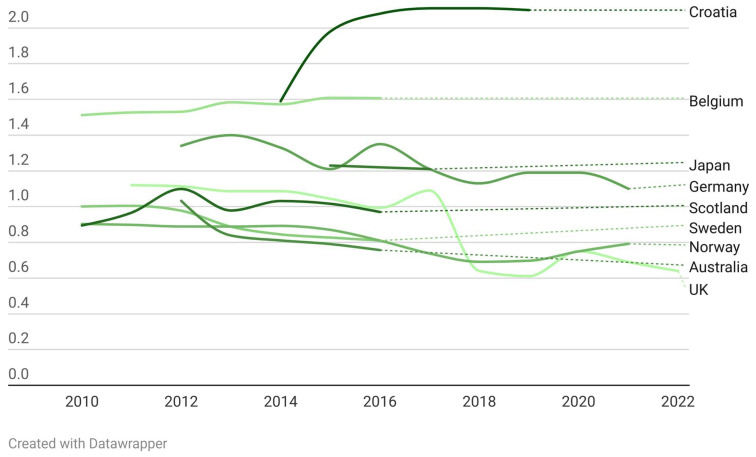
The temporal fluctuations in DID across each nation based on founded data.

Table 2 presents the available DID of antibiotics prescribed by dental practitioners, delineated by country and year. Publicly accessible reports from the English Surveillance Programme for Antimicrobial Utilisation and Resistance (ESPAUR) spanning 2014 to 2021 [30,31,32,33,34] were employed to enrich the data from the United Kingdom (England) in Table 2. This table elucidates both the available and absent data across the surveyed nations. Notably, data for Kosovo and Brazil were limited to single-year observations for 2015 and 2017, respectively, preventing the possibility of trend analysis for these countries. Figure 4 illustrates the temporal fluctuations in DID across each nation. For this visualization, annual DID figures for each country were utilized. South Korea was omitted from this chart due to its data being calculated per 1000 patients. For nations with disparate data points derived from multiple studies (Germany, England, Croatia, and Norway), the highest recorded value was selected for representation.

Within the time period of the founded studies, an upward trend in antibiotic prescriptions by dentists was noted in Croatia, Belgium, and South Korea. Conversely, Sweden, Australia, Scotland, and Japan exhibited a decline. Data from England, Germany, and Norway indicate a consistent decrease in dental antibiotic prescriptions prior to the COVID-19 pandemic, up to the year 2019. However, a surge was observed in 2020, coinciding with the pandemic period. Subsequently, in 2021, England and Germany reported a downturn in these prescriptions, whereas Norway continued to experience an increase. The only data available for the year 2022, originating from England, demonstrate a continued decrease in the DID of antibiotics prescribed by dental professionals in this country (Table 2 and Figure 4).

## 3. Discussion

In this scoping review, a general decline in the volume of antibiotic prescriptions by dental professionals was observed across most surveyed countries over the past decade, with a notable deviation during the COVID-19 pandemic. Contrarily, nations such as Croatia and Belgium exhibited an upward trend, and Norway continued the increasing pattern post-pandemic. The apprehension of virus transmission through aerosols during dental procedures and the proximity between patients and dentists appears to have instigated a reduction in patient visits to dental clinics, consequently prompting a rise in antibiotic prescriptions as a provisional substitute for standard operative dental care [35,36].

Mentioned trends are evident in the monthly antibiotic prescription data from England and Scotland [37,38], which reveal an escalation in prescriptions by dental practitioners concurrent with the peak of restrictions, followed by a modest decline upon the easing of lockdown measures. However, the figures remained elevated compared to pre-pandemic levels. This pattern likely stems from a decrease in routine operative treatments and the subsequent need for more complex interventions due to the postponement of dental services during the lockdown [39]. In contrast, datasets from Spain and Australia [36,39] indicate a reduction in antibiotic prescriptions during the pandemic, followed by an increase once restrictions were lifted. Researchers attribute this to a decline in patient attendance at dental clinics, a reduction in elective procedures such as asymptomatic tooth extractions and implant surgeries, and a rise in self-medication practices among people [36,39].

In the broader context of antibiotic prescription, it is noteworthy that dental practitioners are not the sole prescribers. Surveys indicate that in Australia, dentists are responsible for 3% of all dispensed antibiotic prescriptions [25], while in Norway, they account for a significant 15.6% [9]. In 2016, dentistry-associated prescriptions formed 5.8% of total antibacterial usage in Belgium’s outpatient settings [24]. In the United Kingdom, dental professionals are linked to 10% of antibiotic prescriptions [40], yet they prescribe 3.7% of the total defined daily doses (DDDs) of antibiotics [28]. This highlights the criticality of selecting appropriate metrics for reporting data. Despite the perception that dentists form a minor segment of antibiotic prescribers, Tolksdorf et al. observed that, unlike their outpatient counterparts, the volume of antibiotics prescribed by dentists in Germany has not declined over the past decade [29]. Moreover, dentists in Australia prescribe one out of every six metronidazole and one out of every nine amoxicillin prescriptions [27]. Therefore, our focus is on dental professionals as a key group with the potential to mitigate the prevalence of irrational and inappropriate antibiotic prescriptions.

Survey data indicate that 62% of prescriptions by Indian dentists and 72% of those by Croatian dentists include antibiotics [20,41]. General dentists tend to prescribe antibiotics more frequently than dental surgeons or periodontists [9,42,43]. In Wisconsin, USA, the highest incidence of antibiotic prescriptions among dental specialists was noted in oral surgeons, endodontists, and periodontists [11]. Choi et al. found that in South Korea, dentists are significantly more likely to prescribe antibiotics following tooth extractions in dental hospitals or for multiple teeth extractions, compared to single tooth extractions or in dental clinics [21]. In South Africa, the most common conditions leading to antibiotic prescriptions by dentists were dental abscesses (66%), acute alveolar osteitis (15%), and the removal of impacted third molars (11%), which is similar to the practice among Croatian dentists, where the most frequent indication for systemic antibiotics was periapical abscesses without sinus [5,44].

In the discourse of antimicrobial stewardship within dentistry, it is imperative to acknowledge that, despite the presence of updated guidelines outlining the indications for prophylactic and therapeutic use of antibiotics, observed evidence suggests a substantial proportion of global dental antibiotic prescriptions are nonessential or misaligned with the available guidelines. Illustratively, an examination within the Turkish dental sector unveiled that 96.6% of antibiotic prescriptions were attributed to irrational or uncertain indications [45]. Similarly, Petrac et al. reported that merely less than half (48.31%) of dental antibiotic prescriptions were congruent with established indications [5]. Suda et al.’s initial research in the United States revealed that only 19.1% of antibiotics prescribed for prophylactic objectives from 2011 to 2015 adhered to the guidelines [46]. However, a subsequent publication by the same author indicated a marked improvement, with 72% of prophylactic antibiotics prescribed between 2015 and 2019 deemed necessary, and 91% of cases reflecting appropriate antibiotic selection for the intended prophylactic purposes [47]. This reality underscores the necessity for educational programs and the rigorous evaluation of antibiotic prescription practices in dentistry. Supporting this viewpoint, Kusumoto et al. approved regular educational updates on appropriate antibiotic prescriptions as a strategy to shorten the prevalence of erroneous antibiotic prescriptions within the dental field [12].

To effectively combat antibiotic resistance, countries must develop comprehensive plans to educate and evaluate antibiotic prescriptions across all sectors, with a particular focus on dental settings. These plans should include annual publication of results. For instance, Spain’s national plan against antibiotic resistance, published biennially, assesses antibiotic use in various sectors, including dental clinics, and disseminates updated guidelines to medical and dental centers nationwide [48]. Similarly, in the United Kingdom, the annual ESPAUR report demonstrates the ongoing efforts across the healthcare system to optimize surveillance of antimicrobial use and resistance. It also highlights the implementation of antimicrobial stewardship interventions, including public and professional education and training [28].

### Limitations

The primary limitation of this study was the unavailability and difficulty in accessing updated data. Given that antibiotic resistance is a global issue that transcends national borders [4], it is crucial for each country to publish and make these data accessible to other nations. This would aid the global health system in compiling a comprehensive dataset and devising effective solutions to prevent a potential future crisis. Additionally, it is essential to adopt a uniform metric system for reporting antibiotic usage across all sectors and countries. During our research, we encountered numerous reports from different countries that used varied reporting methods, such as antibiotic prescription rates per dentist [49], per 1000 patients [10], per 1000 population [50,51], or per 1000 dental visits [11].

The DDD system, established by the WHO, offers significant benefits for calculating medication usage. It provides a standardized measure, enabling consistent comparisons of drug usage across different regions and countries. This standardization facilitates trend analysis, allowing for the examination of prescribing patterns and consumption changes over time. Additionally, the DDD system aids in evaluating the impact of interventions, such as regulatory changes or public health campaigns, on drug use. Importantly, the DDD is not related to the actual prescribed daily dose or the number of days a drug is taken; it is a statistical measure representing the assumed average maintenance dose per day for a drug’s main indication in adults [16,52,53,54]. Therefore, it is recommended that data on antibiotic usage in dentistry be published based on the DDDs from each country.

Additionally, our extensive search of available databases yielded 15 publications from 12 countries. However, only 10 of these countries provided continuous datasets for year-to-year comparisons. This reveals a substantial data gap and emphasizes the necessity for further research in this area.

Conversely, our research indicated that the majority of publications utilized data from public centers or individuals covered by insurance. Although this did not introduce any conflicts of interest in the reviewed articles, it poses a risk of selection bias and potential data omission. For instance, in Croatia, 11% of medication prescriptions are issued by private centers [5]. Consequently, relying solely on public datasets could result in the exclusion of at least 11% of relevant data, thereby compromising the comprehensiveness of the analysis.

## 4. Materials and Methods

This section outlines the scoping review conducted to determine the DDDs of antibiotics worldwide. The methodology follows the Preferred Reporting Items for Systematic reviews and Meta-Analyses for Scoping Reviews (PRISMA-ScR) guidelines.

A comprehensive search strategy was employed to gather relevant data from multiple databases. This section details the databases searched, the time frame of the searches, and the specific search terms used.

### 4.1. Search Strategy

A comprehensive search was conducted across several databases, including PubMed, ProQuest, ScienceDirect, Web of Science, Scopus, and EBSCOhost. The initial search took place in April 2024, with a final search to identify newly published articles completed in August 2024. Additionally, emails were sent to authors and companies who had published articles on antibiotic consumption in various countries, and government reports on this topic were also reviewed. The search strategy for PubMed was as follows:

(Antibiotics OR Antibiotic OR Antibacterial OR Anti-Bacterial OR (Anti Bacterial) OR Bacteriocidal OR Bacteriocide OR Bacteriocides OR Anti-Mycobacterial OR (Anti Mycobacterial) OR Antimycobacterial OR (“Anti-Bacterial Agents”[Mesh])) AND ((“dental health services”[MeSH Terms] OR (“dental”[All Fields] AND “health”[All Fields] AND “services”[All Fields]) OR “dental health services”[All Fields] OR “dental”[All Fields] OR “dentally”[All Fields] OR “dentals”[All Fields]) AND (“setting”[All Fields] OR “setting s”[All Fields] OR “settings”[All Fields]) OR “dentistry”[MeSH Terms] OR “dentistry”[All Fields] OR “dentistry s”[All Fields]) AND (“statistics and numerical data”[MeSH Subheading] OR (“statistics”[All Fields] AND “numerical”[All Fields] AND “data”[All Fields]) OR “statistics and numerical data”[All Fields] OR “use”[All Fields]) AND (“prescriptions”[MeSH Terms] OR “prescriptions”[All Fields] OR “prescription”[All Fields]) Filters: from 2000–2024.

For other databases, the search strategy was as follows: (Antibiotic OR antibiotics) AND (Dentistry OR dental OR dental setting OR dentists OR dental surgeon) AND prescription AND DDD Filters: from 2000–2024.

### 4.2. Study Selection

The selection process was conducted by two independent investigators (F.S. and M.M.) and involved both title and abstract screening. During the title screening, articles were initially selected based on their titles, which could be in any language but must have an English or Spanish title. Titles relevant to the research question were selected and imported into EndNote X7 [55]. Duplicate articles were removed using EndNote. During the abstract screening, abstracts were reviewed to identify studies that reported antibiotic prescription rates by dentists or defined daily doses (DDDs) for antibiotics prescribed by dental staff in dental or primary medical centers or provided information to calculate DDDs or antibiotic prescription rates. Discrepancies between the two investigators during these steps were resolved by passing the disputed articles to full-text review.

### 4.3. Inclusion and Exclusion Criteria

During the full-text review of articles, the following criteria served to select included studies to extract data:

- Inclusion Criteria: studies reporting DDDs for antibiotics prescribed by dentists/dental specialists or information provided to calculate DDDs.
- Exclusion Criteria: Survey or questionnaire studies, studies reported solely physicians’ data, single dentist reports, studies based on interns or students’ prescriptions, studies with sample size less than 1000 prescription, and review articles. Review articles were used to manually search their references. These references passed through title and abstract screening stages.

### 4.4. Data Extraction

Selected articles were read in full, and those reporting DDDs per 1000 inhabitants per day (DID) for each year were included in the data extraction and data management step. Articles providing sufficient data to calculate DID, based on the following formula, were also included.

$$\frac{DDDs}{Population\times 365\;}\times 1000=DID$$

For studies that did not specify the DDD or DID for each year, the authors were contacted via email to request the missing information. If no response was received after two attempts, the study was excluded from the analysis.

#### Data Management

Data from the selected articles were compiled into an Excel file prepared by the main investigator (F.S.), including the following variables:

o Name of the first author;
o Title;
o Publication year;
o Time window of study;
o Database to collect data;
o Details of the studied population;
o Care provider (dentists/specialists);
o Most prescribed antibiotics;
o DDDs per 1000 habitants per day (DID) for each year in the time window of the study;
o Type of antibiotic prescription (therapeutic/prophylactic);
o Number of studied prescriptions.

Data extraction was conducted independently by two investigators (F.S and M.M) through reading the full text of the published documents. Discrepancies between the two investigators were resolved through discussion, and data were added based on agreement.

### 4.5. Risk-of-Bias Assessment

The assessment of bias risk in the included studies was conducted utilizing the ROBINS-E tool (Risk Of Bias In Non-randomized Studies—of Exposures [56]). The analysis focused on antibiotic prescriptions as the exposure and the number of DDDs reported as the outcome. For visual representation, a traffic light plot was created to depict the domain-level judgments for each article included in the study. Additionally, weighted bar plots were generated to illustrate the distribution of risk-of-bias judgments across each domain, employing the Robvis tool [57]. It is important to note that the study incorporated one report and one systematic review for which the ROBINS-E tool does not provide a risk-of-bias analysis framework.

Given the increasing criticality of antibiotic resistance over the past decade, this study includes articles reporting DDDs from 2014 onwards. The extracted data and information of the included studies are systematically presented in tabular form. Notably, the DID data for 2016 were the most frequently encountered among the included articles. Consequently, these data were utilized to generate a map using the platform www.datawrapper.de (accessed on 10 August 2024).

## 5. Conclusions

This research identified a slight decreasing trend in the DDD of antibiotics prescribed by dental professionals in the studied countries, with a notable increase during the COVID-19 pandemic. A significant data gap was observed, hindering the ability to compare and present these data globally. This underscores the need for standardized data collection and reporting practices to facilitate comprehensive analysis and comparison across different regions.

## Figures and Tables

**Table 1:** Details of information extracted from included articles.

Author	Country	Time Frame	Database	P/T	D/S	Most Prescribed Antibiotic	Trends
Petrac, L et al. [5]	Croatia	2015–2019	All public health dental offices e-Prescriptions from the Croatian Health Insurance Fund (CHIF).	T	D	Amoxicillin with clavulanic acid (75.2%), amoxicillin (13.8%), clindamycin (4.8%), metronidazole (2.8%) cephalexin (1.4%).	“During observed period, DID increased 6% and 5% in urban and rural areas, respectively”.
Albrecht, H et al. [8]	Germany	2012–2021	Research Institute for Local Health Care Systems (WIdO, Berlin). The report includes all medical and dental prescriptions for members of statutory health insurance (SHI). SHI covers 88% of population.	T	D	The group of penicillin derivatives, consisting of oral penicillin, aminopenicillins, and amoxicillin combinations, accounts for 67.1% of all antibiotics prescribed in 2021. Amoxicillin was the first choice of aminopenicillins.	“An overall decline of 17.9% in the rate of DID in members of the statutory health insurance was observed”.
Tousi, F et al. [9]	Norway	2016–2021	Norwegian Institute of Public Health, Norwegian Prescriptions Database (NorPD). Drugs that are purchased without prescriptions over the counter or supplied to hospitals and nursing homes are not included.	T	D/S	Phenoxymethylpenicillin (68.7%), amoxicillin(10.5%), clindamycin (8.0%), metronidazole (8.5%), erythromycin (1.4%).	“It seems that the impact of the COVID-19 pandemic resulted in the increased use of antibiotic prescriptions compared to an otherwise downward trend”.
ESPAUR [28]	UK	2022	Antimicrobial resistance (AMR) National Action Plan (NAP).	NP	D/S	Amoxicillin (66.7%), metronidazole (28.2%), erythromycin (2.1%).	“Sharp increase observed between 2019 and 2020; DID decreased by 7.4% between 2021 and 2022 but still was more than before pandemic”.
Tolksdorf, K et al. [29]	Germany	2012–2020	German Federal Dental Association (Bundeszahnärztekammer), German state dental associations (Landeszahnärztekammern); all German dental societies as well as the webpage of the German Working Group of the Scientific Medical Societies (Arbeitsgemeinschaft der Wissenschaftlich-Medizinischen Fachgesellschaften, AWMF).	NP	D	Penicillins and aminopenicillins (70% of all prescriptions), clindamycin (26%).	“In contrast to other outpatient physicians, the volume of antibiotics prescribed by dentists in Germany did not decrease over the last decade. Between 2012 and 2015, there was a shift from clindamycin towards amoxicillin”.
Santos, JS et al. [18]	Brazil	2017	Integrated Pharmaceutical Services Management System (SIGAF, in Portuguese)/Secondary data from SIGAF/SES-MG) from the second most populous state in the Brazil.	T	D	Amoxicillin (88.46%), azithromycin (8.89%), amoxicillin with clavulanic acid (3.04%), cephalexin (2.24%).	
Gradl, G et al. [19]	Germany	2017–2021	Data from German Institute for Drug Use Evaluation (DAPI) contain anonymous dispensing data from community pharmacies, claimed to the statutory health insurance (SHI) funds. SHI covers 88% of the population.	NP	D	Amoxicillin (0.63 DID, 50% share), clindamycin (0.29 DID, 24% share), amoxicillin and beta-lactamase inhibitor (0.16 DID, 13% share), phenoxymethylpenicillin (0.08 DID, 6% share), doxycycline (0.03 DID, 2% share), cefuroxime (0.02 DID, 2% share), metronidazole (0.01 DID, 1% share).	“Before the start of the COVID-19 pandemic, DID of all antibiotics showed typical seasonal fluctuations with high values during winter months and low values during summer months. In the period from January 2017 to March 2020, values ranged between 8.92 and 17.93 DID (mean 11.99 ± 2.27). From April 2020 to December 2021, they ranged between 6.32 and 10.86 DID (mean 7.84 ± 1.16)”.
Sutej, I et al. [20]	Croatia	2014–2018	Croatian Health Insurance Fund covering 87.5% of population. The data did not include private prescriptions or medicines dispensed to hospitalized patients.	NP	D	Amoxicillin with clavulanic acid (amoxiclav) (56%), amoxicillin (13.9%), clindamycin (12.5%), metronidazole (10%).	“An increase in the overall prescription rate for all medications prescribed by dentists especially in amoxicillin with clavulanic acid”.
Choi, YY et al. [21]	South Korea	2002–2018	National Health Insurance Corporation (NHIC) of Korea established the National Health Insurance Data Sharing Service (NHISS) database covering more than 99% of population. Ten percent of patients with extraction treatments was selected.	NP	D	Broad-spectrum antibiotics (47.8%).	“The rate of prescribing broad-spectrum antibiotics after tooth extraction was 44.1% in 2002 and 60.4% in 2018 (*p* < 0.001), and it was significantly higher in dental hospitals than in dental clinics (67.4% and 45.9%, respectively, *p* < 0.001)”.
Smith, A et al. [22]	UK	2011–2016	NHS Business Service Authority for England and from the prescribing Information System.	NP	D	Amoxicillin, metronidazole, erythromycin, and phenoxymethyl penicillin (ordered from highest to lowest).	“The highest annual rate of antibiotic prescribing per dentist was in England in 2011 (*n* = 171); by 2016, this had declined to 133 antibiotic prescriptions per year. This represented the highest number of prescriptions per dentist from all 4 countries. In Scotland, the prescription rate per dentist peaked in 2012 (n = 119) and had declined to 87 per annum by 2016. Norway had the lowest rates of prescriptions per dentist (peaking at 31 in 2014) with a decline in prescriptions to 26 per year by 2016. Swedish prescribing levels were highest in 2010 (n = 36) and declined to 28 per annum by 2016”.
	Scotland	2010–2016	NHS National Services Scotland.				
	Sweden		Public Health Agency of Sweden.			Phenoxymethyl penicillin, amoxicillin, clindamycin, and then metronidazole (ordered from highest to lowest).	
	Norway		Norwegian prescription database (NorPD).				
Ono, A et al. [23]	Japan	2015–2017	Database of Health Insurance Claims and Specific Health Checkups of Japan (NDB) covering all citizens and long-term residents.	NP	D	Cephalosporins accounted for the majority of antimicrobials with DID values (proportion of total antimicrobials) of 0.81 (65.6%) in 2015, 0.80 (65.2%) in 2016, and 0.77 (63.7%) in 2017.	“Over the study period, the DID values of penicillin gradually increased while those of cephalosporins slowly decreased”.
Struyf, T et al. [24]	Belgium	2010–2016	Reimbursement data from INAMI-RIZIV, Farmanet covering 98.6% of population. Data of hospitalized patients are not included.	NP	D/S	Broad- or extended-spectrum penicillins such as amoxicillin, with and without an enzyme inhibitor. These two antibiotics accounted for 87.7% of all DID of J01 in 2016.	“After an initial increase from 852 DDD/prescriber in 2010 to 893 DDD/prescriber in 2013, the mean rate of DDD/prescriber declined to 869 in 2016”.
Teoh, L et al. [25]	Australia	2013–2016	Pharmaceutical Benefits Scheme (PBS) covering non-concessional and concessional beneficiaries.	NP	D/S	Amoxicillin (64.3%), metronidazole (13.9%), the broad-spectrum combination product amoxicillin/clavulanic acid (10.4%), clindamycin (5%) in 2016.	“The total number of prescriptions of systemic antibiotics decreased from 2013 to 2016 by 7.3% from 892,483 to 827,020, respectively”.
Haliti N et al. [26]	Kosovo	2015	Twelve primary dental care centers from six administrative regions including Main Family Medicine Center (MFMC) and Family Medicine Center (FMC). One of every fifteen records was chosen for analysis.	NP	D	Co-amoxiclav (J01CR02), with a 1.16 DID, amoxicillin (J01CA04), with a 0.78 DID.	
Ford, PJ et al. [27]	Australia	2001–2012	Australian Government’s subsidized medicine formulary, the Pharmaceutical Benefits Schedule (PBS), covering data of concessional beneficiaries who receive social security benefits.	NP	D	Amoxicillin (66.3%), metronidazole (13.6%), amoxicillin plus clavulanic acid (7.1%), clindamycin (6.1%) in 2012.	“This study shows that dispensed use of antibiotics by dental practitioners in Australia has increased over the studied period”.

P: prophylactic, T: therapeutic, NP: not presented in the article, D: dentists, S: dental specialists.

**Table 2:** Available DID (defined daily doses per 1000 inhabitants per day) of antibiotics prescribed by dental professionals.

Country	2010	2011	2012	2013	2014	2015	2016	2017	2018	2019	2020	2021	2022
England [28,30,31,32,33,34]				1.086	1.086	1.044	0.993	1.09	0.640	0.611	0.75	0.69	0.64
England [22]		1.12	1.1135	1.0814	1.0741	1.0397	0.986						
Brazil [18]								0.0504					
Germany [8]			1.34	1.4	1.33	1.21	1.35	1.21	1.13	1.19	1.19	1.1	
Germany [29]			1.069	1.145	1.106	0.997	1.1168	1.022	0.9596	0.978	0.9877		
Germany [19]								1.20	1.185	1.193	1.169	1.245	
Croatia [5]						1.98	2.08	2.11	2.11	2.1			
Croatia [20]					1.59	1.67	1.73	1.76	1.78				
Norway [9]							0.7796	0.7374	0.691	0.697	0.7494	0.7918	
Norway [22]	0.903	0.8979	0.8887	0.8882	0.8925	0.8699	0.81						
South Korea [21]	6.09	6.25	6.42	6.53	6.59	6.69	6.75	6.85	6.97				
Scotland [22]	0.8945	0.9664	1.0331	0.9782	1.0311	1.0158	0.97						
Sweden [22]	1.0009	1.0044	0.9772	0.8875	0.8446	0.8267	0.81						
Japan [23]						1.23	1.22	1.21					
Belgium [24]	1.512	1.527	1.531	1.584	1.573	1.609	1.607						
Australia [25]				0.8383	0.8105	0.7905	0.7567						
Australia [27]			1.032										
Kosovo [26]						2.17							

## Data Availability

The original contributions presented in the study are included in the article, further inquiries can be directed to the corresponding author.
